# Patient-reported pain and other quality of life domains as prognostic factors for survival in a phase III clinical trial of patients with advanced breast cancer

**DOI:** 10.1186/s12955-016-0449-z

**Published:** 2016-03-25

**Authors:** Emily Nash Smyth, Wei Shen, Lee Bowman, Patrick Peterson, William John, Allen Melemed, Astra M. Liepa

**Affiliations:** Eli Lilly and Company, Lilly Corporate Center, Indianapolis, IN 46285 USA

**Keywords:** Cancer pain, Quality of life, Breast cancer, Prognostic factors, Treatment outcomes

## Abstract

**Background:**

Patient-reported outcomes have been associated with survival in numerous studies across cancer types, including breast cancer. However, the Brief Pain Inventory-Short Form (BPI-SF) and the Rotterdam Symptom Checklist (RSCL) have rarely been investigated in this regard in breast cancer.

**Methods:**

Here we describe a post hoc analysis of the prognostic effect of baseline scores of these instruments on survival in a phase III trial of patients with advanced breast cancer who received gemcitabine plus paclitaxel or paclitaxel alone after anthracycline-based adjuvant or neoadjuvant therapy. The variables for this analysis were baseline BPI-SF “worst pain” and BPI-SF “pain interference” scores, and four RSCL subscales (each transformed to 0–100). Univariate and multivariate Cox models were used, the latter in the presence of 11 demographic/clinical variables. Kaplan-Meier curves and log-rank tests were used to compare survival for patients by BPI-SF or RSCL scores.

**Results:**

Of 529 randomized patients, 286 provided BPI-SF data and 336 provided RSCL data at baseline. Univariate analyses identified BPI-SF worst pain and pain interference (both hazard ratios [HR], 1.07 for a 1-point increase; both *p* ≤ 0.0061) and three of four RSCL subscales [activity level, physical distress, and health-related quality of life (HRQOL) (HR, 0.86–0.91 for 10-point increase all *p* ≤ 0.0104)], to have significant prognostic effect for survival. BPI-SF worst pain (*p* = 0.0342) and RSCL activity level (*p* = 0.0004) were prognostic in the multivariate analysis. Median survival for patients categorized by BPI-SF worst pain score was 23.8 (*n* = 91), 17.9 (*n* = 94) and 14.6 (*n* = 94) months for scores 0, 1–4, and 5–10, respectively (log-rank *p* = 0.0065). Median survival was 23.8 and 14.6 months for patients (*n* = 330) with above- and below-median RSCL activity level scores respectively (log-rank *p* < 0.0001).

**Conclusion:**

Pretreatment BPI-SF worst pain and RSCL activity scores provide distinct prognostic information for survival in patients receiving paclitaxel or gemcitabine plus paclitaxel for advanced breast cancer even after controlling for multiple demographic and clinical factors.

**Trial registration:**

Clinicaltrials.gov, NCT00006459.

## Background

Breast cancer is the most common malignancy worldwide and the largest cause of cancer-related death in women [1]. With advancements across screening and treatment modalities over the past 3 decades, evidence from institutional databases and clinical trials [2, 3] suggests that patients with metastatic breast cancer (MBC) are surviving longer than the median of 18–24 months that has been traditionally reported [4]. However, it unfortunately still remains an incurable disease in nearly all instances, and the goal of treatment is to palliate or prevent symptoms, delay disease progression, and prolong survival [4]. Patients living with advanced disease can experience diminished health-related quality of life (HRQOL) due to cancer- and treatment-related symptoms, such as pain, fatigue, cognitive dysfunction, sleep disturbances, arm morbidity, neuropathy, and menopausal symptoms [5, 6].

Self- report from cancer patients provides a unique perspective that addresses aspects of wellbeing, feelings, and functioning, which may not be otherwise captured with standard clinical assessments [7]. Patients who have reported worsening of symptoms and HRQOL have also experienced deterioration in their clinical condition [7, 8]. Research has shown that HRQOL-related information can serve as a useful adjunct to conventional clinical assessments for oncology patients by improving patient-clinician communication and, in particular, helping clinicians understand patient challenges from a psychosocial perspective [7, 9–16]. Understanding all of these factors is increasingly important in oncology in order to optimize the care of patients [17–20].

Patient-reported outcome data obtained from validated instrumentation have demonstrated consistency and reliability in association with clinical outcomes for cancer and non-cancer conditions [21, 22]. Given the increasing potential for such data to be clinically informative [23, 24], patient-reported outcomes and symptoms have emerged as important measures of cancer treatment in clinical trials. Stratifying patients in clinical trials by baseline HRQOL can result in more homogeneous treatment groups and allow for better understanding of study results [25].

Patient-reported outcomes, assessed via a variety of different instruments, have shown independent prognostic value for survival in post hoc analyses of clinical trials [26–29] and in observational studies of MBC patients undergoing cancer treatment [30, 31]. The prognostic value of HRQOL and associated domains has also been demonstrated in an exploratory fashion across other solid-tumor-specific studies, [7, 32–39] as well as in systematic reviews and meta-analyses [7, 25]. A recent analysis of 7417 patients examined the relative value of different HRQOL domains for multiple cancer types using the European Organisation for Research and Treatment of Cancer (EORTC) Quality of Life Questionnaire-Core 30 (QLQ-C30). Results demonstrated that HRQOL parameters of greatest prognostic value differed by cancer type, and the effect size of each parameter varied according to tumor site. For breast cancer as well as other cancer types, at least one domain provided additional prognostic information to that obtained from clinical and sociodemographic variables [23].

The current report is an exploratory, post hoc analysis of clinical and HRQOL outcomes from a phase III trial comparing gemcitabine plus paclitaxel with paclitaxel alone in patients with advanced breast cancer. As previously reported [40], patients receiving doublet therapy experienced significantly improved survival versus paclitaxel alone and were also more likely to have improved HRQOL over time as measured by the Brief Pain Inventory-Short Form (BPI-SF) and Rotterdam Symptom Checklist (RSCL). Here we evaluate the prognostic effect of BPI-SF and RSCL baseline scores on survival. The BPI-SF is used extensively in clinical practice [41] and it has been used across many studies in breast cancer to assess pain. To our knowledge, however, this is the first exploratory report evaluating the prognostic significance of baseline pain and the relationship to survival utilizing this instrument. Other studies in advanced breast cancer to date have utilized the RSCL [42–44] although none have reported the prognostic significance of baseline scores. The current analysis therefore adds to the body of research by evaluating the prognostic effect of baseline scores for pain and other HRQOL domains on survival in advanced breast cancer utilizing the BPI-SF and the RSCL.

## Methods

### Study design

This was a post hoc analysis of a phase III, multinational, clinical trial evaluating paclitaxel with and without gemcitabine in women with advanced breast cancer who relapsed after adjuvant or neoadjuvant therapy with an anthracycline, or non-anthracycline if there was a contraindication (Clinicaltrials.gov identifier NCT00006459). As reported by Albain et al. [40], eligible patients were to have had Karnofsky Performance Status (KPS) ≥ 70, adequate bone marrow reserve, liver and renal function, normal calcium levels, and an estimated life expectancy of ≥12 weeks at baseline. Prior systemic treatment for metastatic disease was not allowed. Patients were randomly allocated to paclitaxel, with or without concurrent gemcitabine, and treatment cycles continued until disease progression, unacceptable toxicity, or patient withdrawal. There were 529 randomized patients from 19 countries. The primary endpoint was overall survival, which was defined as the time from randomization to death from any cause, and surviving patients were censored at the last visit date. Patients completed the BPI-SF and the RSCL within 1 week prior to randomization and at the end of each treatment cycle. Both the questionnaires were completed by patients for whom validated translations were available. The RSCL was included in this study to better understand the impact of treatment on patient’s symptoms and the influence of symptoms on physical and psychological function. The BPI-SF was included to allow a more focused evaluation of pain, which is not specifically evaluated by the RSCL. Efficacy, safety, and patient-reported outcomes results have been previously reported [40, 45, 46]. The study was conducted according to Declaration of Helsinki and good clinical practice guidelines, and was approved by each participating center’s ethics review board. All patients signed informed consent forms that described the clinical and HRQOL components of the study.

### Patient-reported outcome instrumentation

The BPI-SF is a valid and reliable instrument [41], originally developed to assess chronic pain and its impact on HRQOL [47]. This instrument is widely used in clinical practice settings and research to measure the impact of cancer pain [41]. Patients rate their pain and its effects as experienced in the preceding 24 hours. The BPI-SF measures pain intensity (worst, least, average, current), pain relief, and interference of pain (on 7 HRQOL dimensions of general activity, mood, walking, normal work, relations with others, sleep, and enjoyment of life). Scores for the worst pain item as well as pain interference were used in this analysis. Worst pain scores range from 0 (no pain) to 10 (worst pain). Pain interference scores range from 0 (no interference) to 10 (complete interference) and were calculated as the mean of the seven pain interference items.

The RSCL is a validated instrument for measuring HRQOL and symptoms in cancer patients [48, 49]. The instrument assesses symptoms and effects of symptoms on anxiety, depression, functional status, and quality of life during the week prior to completing the questionnaire. It has been used previously by patients with advanced breast cancer [44, 50], as well as in numerous other cancer trials [51–55]. Patients answer items for different symptoms via a Likert scale (not at all, a little, quite a bit, very much) comprising four dimensions: physical distress (23 items), psychological distress (7 items), activity level (8 items), and a single item to measure overall HRQOL. For the purposes of this study, the RSCL subscales were each transformed to 0–100, with 100 as the best score.

### Statistical analyses

The present post hoc analysis utilized patient-reported outcome scores measured at baseline only. All patients with a baseline BPI-SF or RSCL assessment were included, and data from the two study treatment arms were combined for all analyses.

The associations between baseline measures of clinician-assessed KPS and patient-assessed BPI-SF and RSCL subscale scores were analyzed using one-way analyses of variance (ANOVA). Univariate Cox proportional hazards models were used to determine the prognostic effect of each variable on survival. Multivariate Cox proportional hazard models were used to determine the prognostic effect of each RSCL and BPI-SF score in the presence of 11 baseline demographic and clinical prognostic factors that had been used in the clinical study for randomization stratification or preplanned subgroup analyses. In general, a finding of an effect in a univariate analysis is sufficient to show that the subscale provides prognostic information for survival; the same finding in a multivariate analysis suggests that the prognostic value is additional to what could be obtained from demographic and clinical characteristics. For the current analysis, all were assessed as binary categorical variables: KPS (<90 vs ≥90), estrogen receptor status (positive vs negative), progesterone receptor status (positive vs negative), presence of visceral disease (yes vs no), age (<65 vs ≥65), prior radiotherapy (yes vs no), prior hormonal treatment (yes vs no), menopausal status (pre- or peri-menopausal vs post-menopausal), basis for pathologic diagnosis (histological vs cytological), race (Caucasian vs non-Caucasian), and pathologic diagnosis (ductal vs non-ductal). Kaplan-Meier curves and log-rank tests were used to compare survival among patient groups categorized into three and two categories for the BPI-SF [56] and the RSCL respectively. The categories of the BPI-SF were divided into approximately equal numbers of patients: worst pain scores (0 = no pain, 1 to 4 = mild pain, 5 to 10 = moderate/severe pain), pain interference scores (0 to <0.5 = no interference, ≥0.5 to <4.5 = mild interference, ≥4.5 to ≤10 = moderate/severe interference). Dichotomized patient groups (>median, ≤ median) were evaluated for each of the four RSCL subscale scores; a choice of baseline median as the cut point was made by the study team so as to evaluate similar numbers of patients above and below the median. Statistical significance was defined as *p* ≤ 0.05, and no adjustment was made for multiplicity. All statistical analyses were conducted using SAS® version 9.2 or later version.

## Results

### Patient population and baseline characteristics

Table 1 shows the baseline demographic and disease characteristics of patients who contributed data to this analysis. This analysis included all patients who completed the BPI-SF (*n* = 286) and RSCL (*n* = 336) questionnaires at baseline. Approximately one-third to one-half of patients in the original study did not complete these assessments primarily due to a lack of a validated translation of the questionnaire in their language [46]. Patient demographic and clinical characteristics were comparable between those who completed the BPI-SF or the RSCL, with the majority of patients less than 65 years of age, post-menopausal, Caucasian, and having metastatic disease at diagnosis. Additionally, similar proportions of patients in each group were estrogen or progesterone receptor positive or negative, had ductal or non-ductal breast carcinoma, and received prior radiotherapy or hormonal therapy. The number of patients eligible to complete the RSCL based on validated translations was 368. However, 32 patients did not complete the RSCL at baseline due to failure by the site to administer (*n* = 11), patient refusal (*n* = 11), or other reasons, the specifics of which were not documented (*n* = 10). The number of patients eligible to complete the BPI based on validated translations was 308. Of these, 22 patients did not complete the BPI at baseline due to failure by the site to administer (*n* = 8), patient refusal (*n* = 9), or other reasons (*n* = 5).

**Table 1:** Baseline Demographic and Disease Characteristics for Patients Who Provided BPI-SF or RSCL at Baseline

	BPI-SF patients	RSCL patients
Number of patients with baseline assessment	286	336
Age, median (range)	55 (27–83)	54 (27–83)
Age ≥ 65 years (*n*, %)	56 (19.6)	59 (17.6)
Ethnicity, *n* (%)		
Caucasian	202 (70.6)	220 (65.5)
Asian	15 (5.2)	16 (4.8)
Hispanic	55 (19.2)	86 (25.6)
Other	14 (4.9)	14 (4.2)
Stage at entry (*n*, %)		
Unresectable	14 (4.9)	15 (4.5)
Metastatic	272 (95.1)	321 (95.5)
Karnofsky Performance Status (*n*, %)		
< 90	75 (26.2)	86 (25.6)
90 or 100	210 (73.4)	249 (74.1)
Unknown	1 (0.3)	1 (0.3)
Tumor metastatic site (*n*, %)		
Visceral	213 (74.5)	251 (74.7)
Non-visceral only	73 (25.5)	85 (25.3)
Menopausal status		
Peri-menopausal	10 (3.6)	12 (3.6)
Post-menopausal	237 (84.3)	277 (83.7)
Pre-menopausal	34 (12.1)	42 (12.7)
Unknown	5 (1.7)	5 (1.5)
Estrogen receptor status (*n*, %)		
Positive	117 (40.9)	133 (39.6)
Negative	120 (42.0)	141 (42.0)
Unknown	49 (17.1)	62 (18.5)
Progesterone receptor status		
Positive	101 (35.3)	113 (33.6)
Negative	123 (43.0)	146 (43.5)
Unknown	62 (21.7)	77 (22.9)
Pathologic diagnosis		
Ductal breast carcinoma	228 (79.7)	276 (82.1)
Non-ductal breast carcinoma	58 (20.3)	60 (17.9)
Basis for pathologic diagnosis		
Histopathological	257 (89.9)	303 (90.2)
Cytological	29 (10.1)	33 (9.8)
Prior radiotherapy		
Yes	196 (68.5)	238 (70.8)
No	90 (31.5)	98 (29.2)
Prior hormone therapy		
Yes	151 (52.8)	164 (48.8)
No	135 (47.2)	172 (51.2)

*BPI-SF* Brief Pain Inventory-Short Form, *RSCL* Rotterdam Symptom Checklist

### Association of the BPI-SF and RSCL Scores with KPS

Given the role of clinician-assessed performance status as a clinical measure in these patients, we sought to specifically characterize the association between patient-assessed BPI-SF and RSCL versus KPS. Both BPI-SF variables (pain interference and worst pain) were significantly associated with KPS (pain interference *p* ≤ 0.0001; worst pain *p* = 0.0030). Patients with better performance status (higher KPS) had less pain interference and less intensity of worst pain (Table 2). There was a positive association between mean RSCL subscale scores and KPS for activity level, physical distress, and overall HRQOL, indicating that patients with better performance status had greater levels of activity and HRQOL as well as lower levels of physical distress (all *p* < 0.0001, Table 3). While there was a positive trend in RSCL scores with increasing KPS, there was no significant association between KPS and the psychological distress subscale (*p* = 0.1630).

**Table 2:** Mean BPI-SF^a^ by KPS

KPS	BPI-SF – Pain Interference		BPI-SF – Worst Pain	
	*n*	Mean (SD)	*n*	Mean (SD)
Overall^b^	281	2.2 (2.63)	278	3.2 (3.03)
≤70	23	4.6 (3.67)	23	4.6 (3.60)
80	51	2.8 (2.75)	49	4.1 (2.82)
90	100	1.9 (2.38)	100	3.3 (2.88)
100	107	1.6 (2.19)	106	2.5 (2.94)
		*p* < 0.0001^c^		*p* = 0.0030^c^

*BPI-SF* Brief Pain Inventory-Short Form, *KPS* Karnofsky Performance Status, *SD* standard deviation

^a^BPI-SF item scores range from 0 (no pain or interference with daily living) to 10

^b^Overall patient numbers < 286 because not all answered every BPI-SF item ^c^ *P*-values derived from one-way analysis of variance

**Table 3:** Mean RSCL^a^ Subscale Scores by KPS

KPS	RSCL – Activity		RSCL – Physical		RSCL – Psych		RSCL – HRQOL	
	*n*	Mean (SD)	*n*	Mean (SD)	*n*	Mean (SD)	*n*	Mean (SD)
Overall^b^	329	83.3 (22.65)	333	83.5 (12.37)	332	64.4 (23.76)	323	66.0 (24.14)
≤70	25	51.2 (27.11)	26	74.4 (14.21)	26	56.5 (26.78)	26	38.5 (28.19)
80	58	71.7 (26.25)	59	78.5 (12.27)	58	60.3 (23.31)	58	58.6 (22.13)
90	110	85.2 (18.62)	111	84.9 (11.75)	111	65.1 (24.19)	109	67.1 (22.39)
100	136	92.7 (14.32)	137	86.2 (11.17)	137	67.1 (22.67)	130	74.0 (20.54)
		*p* = 0.0001^c^		*p* < 0.0001^c^		*p* = 0.1630^c^		*p* < 0.0001^c^

Activity: Activity level; Physical: Physical distress; Psych: Psychological distress

*HRQOL* health-related quality of life; *KPS* Karnofsky Performance Status; *RSCL* Rotterdam Symptom Checklist; *SD* standard deviation

^a^ RSCL scores transformed to a range from 0 to 100, with 100 as best score

^b^ Overall patient numbers < 336 because not all answered every RSCL item

^c^ *p*-values derived from one-way analysis of variance

### Prognostic Effect of BPI-SF and RSCL on Survival

In the univariate analyses, patients with higher BPI-SF scores (indicating worse pain or pain interference) had lower survival rates. In the case of the RSCL, patients with higher scores (indicating lower symptom burden) had better survival. Significant prognostic effects on survival were observed for baseline scores of both BPI-SF worst pain and pain interference (both hazard ratios [HRs], 1.07 for 1-point increase; both *p* ≤ 0.0061) and three of the four RSCL subscales (activity level, physical distress, and overall HRQOL) (HR, 0.86–0.91 for 10-point increase; all *p* ≤ 0.0104; Table 4). Psychological distress had no significant prognostic effect on survival.

**Table 4:** Prognostic Effect of BPI-SF and RSCL on Survival

HRQOL subscale variables ^a^	*n* ^b^	HR	95 % CI	*p*-value	HR	95 % CI	*p*-value
		Univariate			Multivariate^c^		
BPI-SF – Pain Interference	282	1.07	1.02–1.12	0.0061	1.02	0.97–1.08	0.3931
BPI-SF – Worst Pain	279	1.07	1.03–1.12	0.0013	1.05	1.00–1.10	0.0342
RSCL – Activity Level	330	0.86	0.82–0.91	<0.0001	0.89	0.83–0.95	0.0004
RSCL – Physical Distress	333	0.88	0.81–0.97	0.0104	0.92	0.83–1.02	0.1053
RSCL – Psychological Distress	332	0.96	0.91–1.01	0.1025	0.98	0.92–1.03	0.3800
RSCL – HRQOL	324	0.91	0.87–0.96	0.0002	0.95	0.90–1.01	0.0810

*BPI-SF* Brief Pain Inventory-Short Form; *CI* confidence interval; *HR* hazard ratio; *HRQOL* health-related quality of life; *KPS* Karnofsky Performance Status; *n* number of patients with nonmissing values on a variable among randomized patients; *RSCL* Rotterdam Symptom Checklist

^a^HRs for every 1-point increase in the BPI-SF and every 10-point increase in the RSCL subscales

^b^Includes 1 patient with missing KPS score and not included in Tables 2 and 3. This patient’s scores were as follows: BPI Pain Interference, 0; BPI Worst Pain, 0; RSCL Activity, 100; RSCL Physical, missing; RSCL Psychological, missing; RSCL HRQOL, 83.33

^c^Multivariate Cox proportional hazards models were used to determine each HR in the presence of 11 demographic/clinical variables: age, race, KPS, estrogen receptor status, progesterone receptor status, presence of visceral disease, prior radiotherapy, prior hormonal treatment, menopausal status, basis for pathological diagnosis, and pathological diagnosis

In the multivariate analyses, the BPI-SF worst pain item and the RSCL activity level remained significant prognostic factors (*p* = 0.0342, *p* = 0.0004, respectively; Table 4) in the presence of the 11 baseline demographic or clinical variables. The pain interference subscale, physical distress, and overall HRQOL scores were no longer significant. Consistent with the univariate analysis, psychological distress was not prognostic for survival.

### Survival Time by BPI-Worst Pain, Pain Interference, and RSCL Categories

**Fig. 1:**
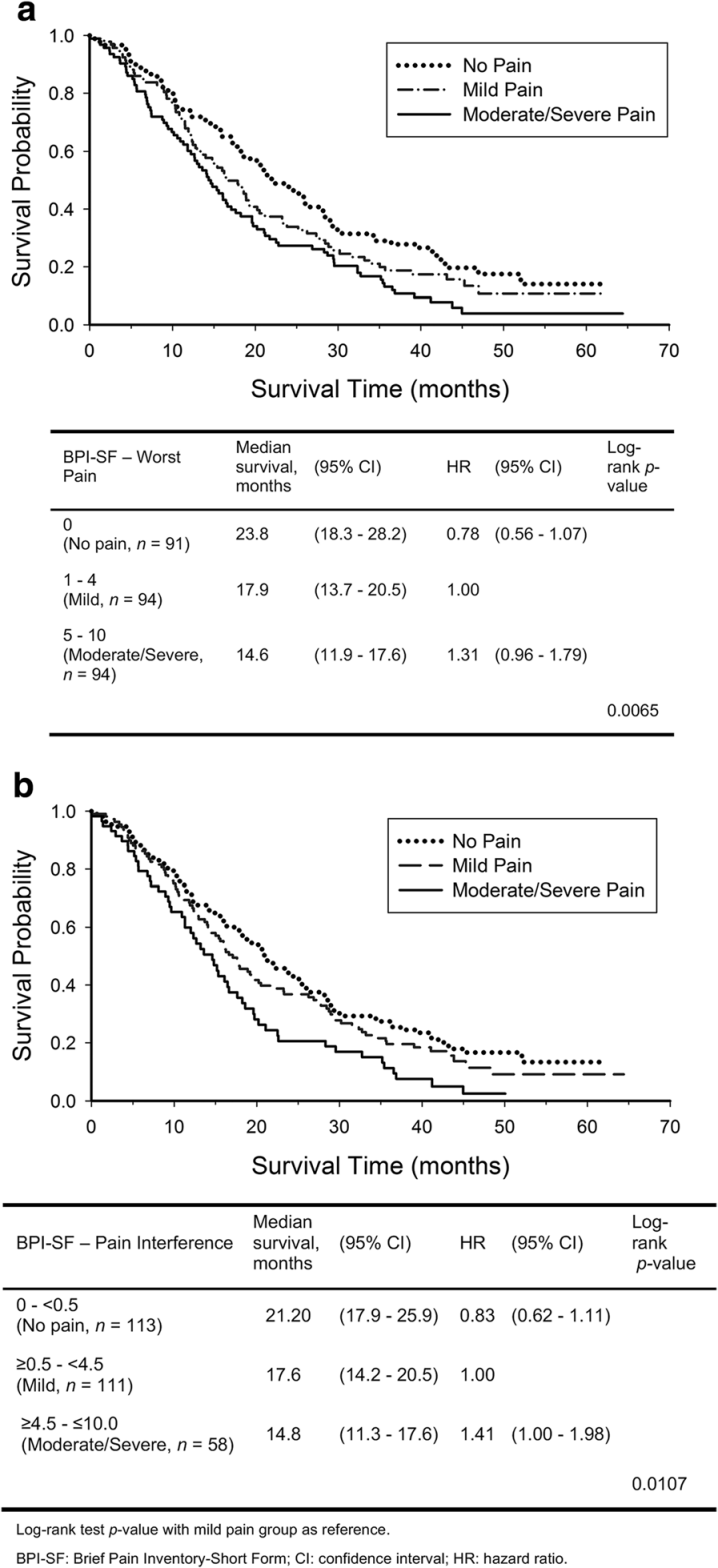
Kaplan-Meier Survival Curves by BPI-Worst Pain Categories. **a** Worst Pain **b** Pain Interference

**Fig. 2:**
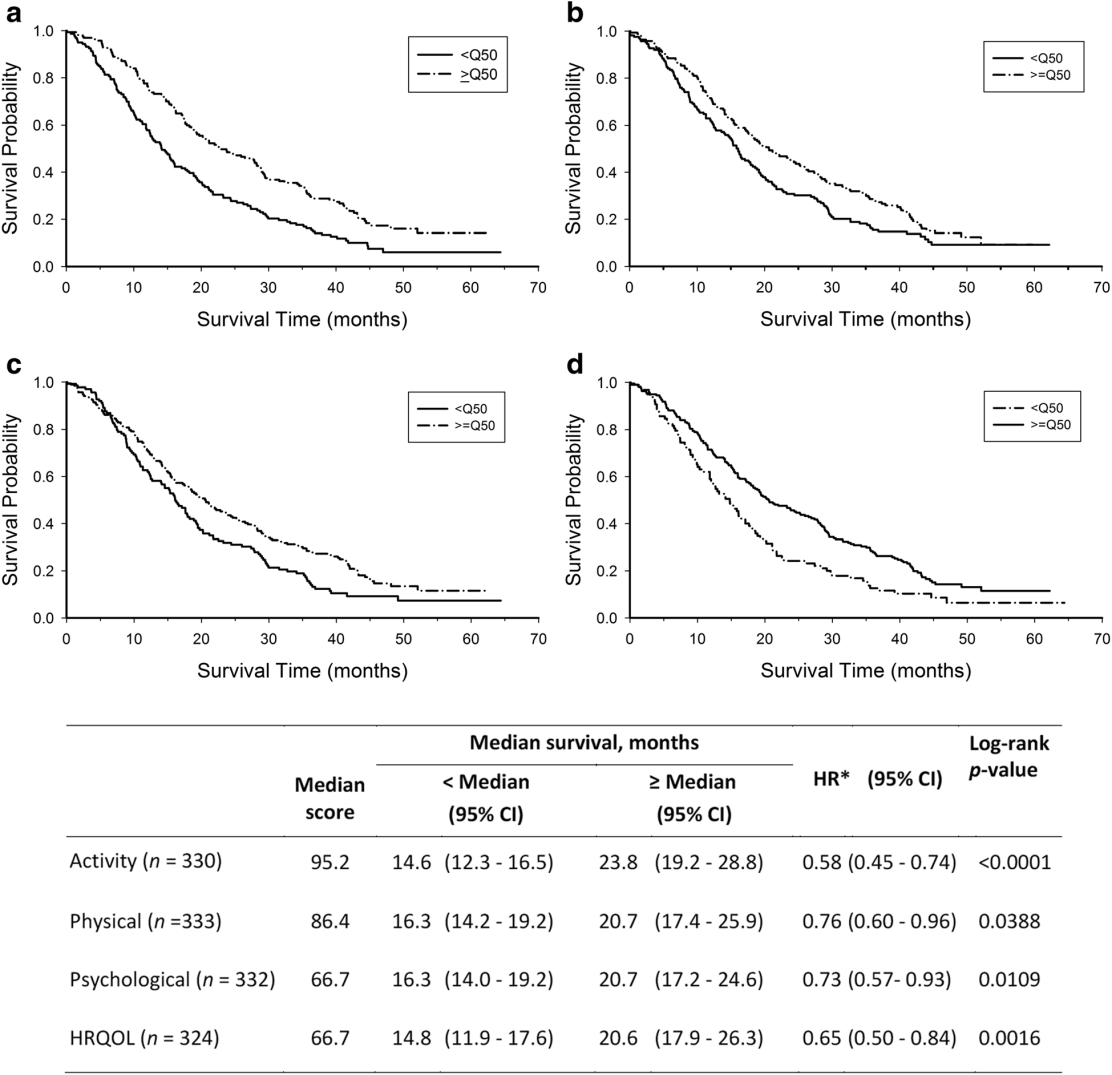
Kaplan-Meier Survival Curves by Rotterdam Symptom Checklist (RSCL) Categories. **a**. Activity Level. **b**. Physical Distress. **c**. Psychological Distress. **d**. Overall Health-Related Quality of Life (HRQOL). * Multivariate Cox proportional hazards models were used to determine each hazard ratio (HR) in the presence of 11 demographic/clinical variables: age, race, KPS, estrogen receptor status, progesterone receptor status, presence of visceral disease, prior radiotherapy, prior hormonal treatment, menopausal status, basis for pathological diagnosis, and pathological diagnosis. Reference is the < median group; Q50 is the median of each RSCL subscale

The median survival time for patients with a BPI-SF worst pain score of 0 (no pain) was 23.8 months (*n* = 91), versus 17.9 months (*n* = 94) for scores 1–4, and 14.6 months (*n* = 94) for scores 5–10 (log-rank *p* = 0.0065, Fig. 1a). Median survival time for patients with BPI-SF pain interference scores was 21.2 months (*n* = 113) (no pain; scores 0 to <0.5) versus 17.6 months (*n* = 111) (scores ≥0.5 to <4.5) and 14.8 months (*n* = 58) (scores ≥4.5 to ≤10; log-rank *p* = 0.0107; Fig. 1b). The median survival time was 23.8 months for patients with median (95.2) or greater activity level scores versus 14.6 months for patients with scores below median (log-rank *p* < 0.0001, Fig. 2). Similarly, for the activity, physical, psychological, and HRQOL subscales, patients with median or greater scores had significantly better survival (log-rank *p* < 0.05, Fig. 2).

## Discussion

We have shown in this post hoc analysis that baseline patient-reported outcomes and symptoms, as measured by the BPI-SF and RSCL, were prognostic for survival in patients with locally recurrent or metastatic breast carcinoma who received gemcitabine with or without paclitaxel. The use of two different, validated instruments was informative when exploring this prognostic effect in that they measure distinct aspects of HRQOL in cancer patients [57], and the present study allowed for a model-dependent sensitivity analysis.

In this study, the worst pain and pain interference measures of the BPI-SF as well as activity level, physical distress, and HRQOL scores of the RSCL were all prognostic for survival in the univariate analysis. The BPI-SF worst pain and RSCL activity subscales retained a statistically significant effect in the multivariate analysis, which incorporated clinical, and sociodemographic variables commonly used as prognostic indicators in advanced breast cancer (Table 4). Clinical factors previously reported as prognostic via multivariate analysis for this study cohort included time from diagnosis to randomization, number of tumor sites, estrogen receptor status, and KPS [40].

The RSCL activity subscale had the greatest effect on survival, as reflected by the hazard ratio in the univariate analysis (Table 4). In addition, it was the only subscale to retain a statistically significant effect in the multivariate analysis that considered the other RSCL subscales as well as baseline clinical or demographic characteristics. The RSCL psychological distress subscale had no relationship with survival based on 10-point increments in a Cox proportional hazards model (Table 4). However, the dichotomous comparison (above and below median scores) did reveal a significant difference in survival curves (log-rank *p* = 0.0109; Fig. 2). It is important to note that systematic literature reviews have found only minimal evidence for prognostic effects of psychosocial HRQOL domains on cancer survival [7, 58].

The most commonly utilized instruments in MBC trials include the Functional Assessment of Cancer Therapy-Breast (FACT-B) and the EORTC QLQ-C30 with or without the EORTC breast cancer-specific module, QLQ-BR23 [59]; positive prognostic results have been demonstrated in previously reported analyses of advanced breast cancer that used baseline EORTC QLC-C30 assessments [27–29].

The current exploratory analysis of a clinical study population further adds to the body of evidence that patient-reported outcomes and symptoms can be prognostic indicators in patients with advanced breast cancer and as such, a useful adjunct for informing the routine care of patients. It is important to note that because patient-reported responses can be subject to social and environmental factors, this information is best used as an added tool to all other demographic and clinical considerations that are part of patient care [9]. The reasons for the relationship between patient-reported outcomes and symptoms, and survival have not been completely elucidated. However, it is thought that patient-reported outcomes may be sensitive to disease progression or markers of patient behaviors or other characteristics, such as treatment adherence or healthy lifestyles [7]. In addition, the inclusion of multiple items in patient survey instruments may enhance their diagnostic sensitivity whether in clinical management or in oncology drug trials [7].

It is important to reiterate that the current study was a post hoc analysis of HRQOL data originally recorded as secondary outcomes in a prospective clinical trial [46]. Although not all randomized patients were included in the analyses due to limited availability of translations of the BPI-SF and RSCL, a high percentage of the patients with translations available to them completed the questionnaires; furthermore because only baseline questionnaire responses were used, there was less potential for nonrandom missing data. Prognostic factors in the current study were not defined prior to study enrollment, and no adjustments were made for multiplicity. Therefore, it would be worthwhile to evaluate the hypothesis prospectively in order to further support the utility of patient-survey instruments for the added benefit of assessing patient prognosis both in clinical trials as well as in routine clinical practice. If confirmed, these data could be used to potentially identify appropriate cut points for the purposes of prognostic evaluation and study stratification. It is important to reiterate that these findings are specific to first-line advanced disease and cannot be generalized to other lines of therapy. Furthermore, results discordant with those of other studies may have been due to differences in statistical techniques or instrumentation.

## Conclusion

In conclusion, this post hoc analysis of patients with advanced breast cancer showed that pretreatment BPI-SF worst pain and RSCL activity scores provide additional prognostic information for survival beyond that available from standard demographic and clinical characteristics. Our findings further support the concept that patient-reported outcomes can be useful additional prognostic factors in advanced breast cancer and help guide effective clinical decision making by revealing manifestations of malignant disease not systematically evaluated by formal clinical measures.

## Abbreviations

BPI-SF: Brief Pain Inventory Short Form
EORTC QLQ-C30: European Organisation for Research and Treatment of Cancer Quality of Life Questionnaire Core 30
HR: hazard ratio.
HRQOL: health related quality of life
KPS: Karnofsky Performance Status
MBC: metastatic breast cancer
RSCL: Rotterdam Symptom Checklist
